# Trends in performance of the National Measles Case-Based Surveillance System, Ministry of Health and Child Welfare, Zimbabwe (1999 - 2008)

**Published:** 2012-01-11

**Authors:** Regis Choto, Addmore Chadambuka, Gerald Shambira, Notion Gombe, Mufuta Tshimanga, Stanley Midzi, Joseph Mberikunashe

**Affiliations:** 1 MPH Programme, Department of Community Medicine, University of Zimbabwe, Zimbabwe; 2 Department of Epidemiology and Disease Control, Ministry of Health and Child Welfare, Harare, Zimbabwe

**Keywords:** Diseases surveillance, active surveillance, indicators, Measles, Zimbabwe

## Abstract

**Introduction:**

Since adoption of the measles case-based surveillance system in Zimbabwe in 1998, data has been routinely collected at all levels of the health delivery system and sent to national level with little or no documented evidence of use to identify risky populations, monitor impact of interventions and measure progress towards achieving measles elimination. We analysed this data to determine trends in the national measles case-based surveillance system (NMCBSS).

**Methods:**

A retrospective record review of the NMCBSS dataset for period 1999 –2008 was conducted, assessing trends in proportions of investigated cases; timeliness and nature of specimens received at laboratory; timeliness of feedback of serology results, proportion of cases confirmed as measles and national annualized rates of investigation. Comparisons with WHO performance indicators were done. The secondary data analysis was done in Excel and Epi-Info statistical software.

**Results:**

Cumulatively 4994 suspected cases were reported and investigated between 1999 and 2008. Reported suspected and confirmed measles cases declined from 24, 5% and 5.9% respectively in 2000 to 3.9% and 1.0% respectively in 2008. Proportion of cases with blood specimens collected and proportion reaching laboratory timely increased from 83% and 65% respectively in 1999, to 100% and 82% respectively in 2008. Proportion of specimens arriving at laboratory in good condition improved from 65% in 2004 to 94% in 2008 while timeliness of feedback of serology results improved from 4% in 2004 to 65% in 2008. Sensitivity of the NMCBSS however has been weakening, declining from 9.04 cases investigated per 100 000 population per year in 2000 to 1.58 cases/100 000/year in 2008.

**Conclusion:**

The NMCBSS improved in quality, timeliness and feedback of laboratory results of specimens sent for investigation, but its sensitivity declined mainly due to reduced capacity to detect and confirm measles cases. We recommend training staff on active surveillance of cases and more support and supervisory visits to strengthen EPI surveillance.

## Introduction

Public health surveillance has been defined as the ongoing, systematic collection, analysis, interpretation and dissemination of data regarding a health related event for use in public health action to reduce mortality and to improve health [1–3]. Data generated from such public health surveillance systems is used for guiding immediate public health action, program planning and evaluation, monitor trends in the burden of disease and formulating research hypotheses. As an essential to public health action and for surveillance purposes, the public health information systems involved need to include a variety of data sources [4]. These systems vary from a simple system collecting data from a single source to electronic systems that receive data from many sources in multiple formats, to complex surveys. The number and variety of systems, will likely increase with advances in electronic data interchange, and integration of data which will also heighten the importance of patient privacy, data confidentiality and system security. To ensure that problems of public health importance are monitored effectively and efficiently, periodic evaluations of public health surveillance systems are critical. Despite achieving and sustaining global measles vaccination coverage of about 80% over the past decade, worldwide measles remains the fifth leading cause of mortality among children aged less than 5 years [5]. It accounts for more than 30 million cases annually and 0.9 million deaths every year, with approximately half of these occurring in Africa [6]. Measles is vaccine preventable and a child is eligible for measles vaccine once they attain nine months of age. Measles is one of the diseases earmarked for elimination worldwide. In line with the WHO pre-elimination goal for measles, the WHO African Region recommended to its member countries the measles case based surveillance with laboratory confirmation as one of the main strategies for measles control. Under this case based surveillance system, every measles case is reported and investigated immediately and also included in weekly reporting system. In countries where the main objective is to completely interrupt measles transmission, an intensive case-based surveillance to detect, investigate and confirm every suspected measles case in the community is recommended. To achieve measles pre-elimination, a high routine vaccine coverage rate (with at least 90% of infants in all regions needing vaccination before their first birthday) and periodic vaccination supplemental immunization activities (reaching at least 95% of the targeted population) are required. The periodicity of supplemental immunization activities (SIAs) though routinely coming every 3 to 4 years, is determined by the routine EPI coverage, the coverage during catch up SIAs and the results of measles surveillance.

Measles elimination is defined as the absence of endemic measles cases for a period of twelve months or more, in the presence of adequate surveillance, and when the following criteria are met: (i) achieving and maintaining at least 95% coverage with both first dose measles vaccination and the second opportunity of measles vaccination in all districts and nationally; (ii) having less than 10 confirmed cases in 80% or more of measles outbreaks; (iii) achieving a measles incidence of < 1 confirmed measles case per million population per year [7]. In Zimbabwe, measles surveillance is in the elimination phase and as such a measles case-based surveillance system was adopted since 1998, with objectives to identify at risk populations; monitor impact of routine and supplementary immunization activities; measure progress towards achieving elimination of measles and monitor accumulation of susceptible populations and take appropriate action. In line with the WHO AFRO regional strategy, Zimbabwe commenced supplemental immunization activities in 1998 with follow up SIAs being done in 2002, 2006, and 2009. These SIAs were targeting children 9 months to 14 years in catch-up campaigns and 9 months to 5 years during periodic follow-up campaigns.

Since the adoption of the measles case-based surveillance system in 1998, in Zimbabwe, data has been routinely collected at all levels of the health delivery system and sent to the national level. To our knowledge there was no documented evidence to show data collected was periodically being used to identify at risk populations, monitor impact of interventions and measure progress towards achieving measles elimination. We analysed this data to determine trends in performance of the national measles case-based surveillance system and advise policy.

## Methods

### Surveillance system

In Zimbabwe, surveillance data for suspected measles cases is routinely collected at all levels of the health delivery system using the measles case based surveillance form and sent to MOHCW Head Office. Data from the primary facilities is sent through the district, province onto national level where it is entered into the measles case-based surveillance system database, (EPI INFO based), where it is consolidated, cleaned, analyzed and disseminated for action. A copy of this database is also shared with the World Health Organisation.

Important variables captured by the dataset include; patient's name, age, sex, vaccination status, area of residence (by province, district), date of rash onset, date of first investigation (notification, specimen collection and specimen sending), dates of specimen arrival and dispatch of results from the national virology laboratory, specimen condition on arrival at national virology laboratory, Measles IgM test result, Rubella IgM test and final case classification. Feedback of the final case classification is given to the reporting facility through the same channel used for reporting.

### Surveillance Methods

We conducted a retrospective record review of the national measles case-based surveillance dataset for the period 1999 to 2008, assessing for trends in proportion of investigated cases; timeliness and nature of specimens received at laboratory; timeliness of feedback of serology results, proportion of cases confirmed as measles and national annualized rates of investigation. Comparisons with WHO recommended performance indicators (Table 1) to determine quality of measles surveillance were done and secondary data analysis was done after cleaning, using Microsoft Excel and Epi-Info to generate frequencies and proportions.

**Table 1:** Indicators of Quality of Measles Surveillance

Indicators of quality of measles surveillance [8]	Target
Proportion of reported suspected measles cases from whom blood specimens have been collected (excluding epidemiologically linked cases from the denominator)*	≥80%
Proportion of districts that have reported at least 2 suspected cases of measles with a blood specimen per year*	≥80%
Annualized rate of investigation (with blood specimens) of suspected measles cases	>2.0 per 100,000 population
Proportion of measles outbreaks investigated with blood specimens from the first five cases	≥80%
Proportion of suspected measles case investigated within 3 days following notification	≥80%
Proportion of serum/ dried blood specimens arriving at lab within 3 days of being taken	≥80%
Proportion of laboratory confirmed measles cases	<10%
Proportion of feedback of serology results sent from the laboratory to the national level within 7 days of receipt of specimens at the lab**	≥80%
Proportion of serum specimens arriving at the national measles laboratory in good condition** ( i.e. adequate volume, no leakage, not turbid, not desiccated)	>90%
Proportion of representative 2 serum specimens sent quarterly by the national laboratories to the regional reference labs for re-confirmation as part of quality assurance measures**	>10%
Proportion of concordance of measles IgM results between the national measles lab and the regional reference lab**	>90%

* Main surveillance performance indicators;

** Measles laboratory performance indicators

Permission to proceed with the study was obtained from the Director of Epidemiology and Disease Control, Ministry of Health and Child Welfare and Health Studies Office. Ethical clearance was obtained from Medical Research Council of Zimbabwe. Names of patients and addresses were omitted from the analysis. Confidentiality was assured and maintained.

## Results

Cumulatively 4994 suspected measles cases were reported and investigated for the period January 1999 to December 2008. Out of these, 200 cases (4.0%) had their records duplicated, which resulted in a 4.4% over estimation of reported cases. Among the 4994 cases investigated, the 1-4 year and 5-14 year age groups were the most affected accounting for 1173 (23.5%) and 2 668 (53.4%) of the cases respectively. The minority of reported cases came from the below 1 year and above 15 years age groups which accounted for 157 (3.1%) and 116 (2.3%) cases respectively. One hundred and three cases (18.1%) of the total reported cases had missing information on age group.

The proportion of males and females among the cases reported for the period 1999-2008 was almost similar with a male to female distribution ratio of (2306: 2184) of 1:1. Five hundred and four cases (10.1%) had missing information on gender status. Fifty percent (2579 cases) of the overall suspected cases investigated had received at least a single dose of measles vaccine and a very small proportion 0.5% (24 cases) had not received any measles vaccination before. However, 2139 cases (42.8%) had missing information on vaccination status. Out of the 4994 cumulative suspected measles cases reported, 150 cases were confirmed as measles and of these, 5 cases (3.3%) had not been vaccinated before and 53 cases (35.3%) had at least received a single dose of the measles vaccine. The remaining 71 cases (51.3%) had missing information on vaccination status.

**Figure 1:**
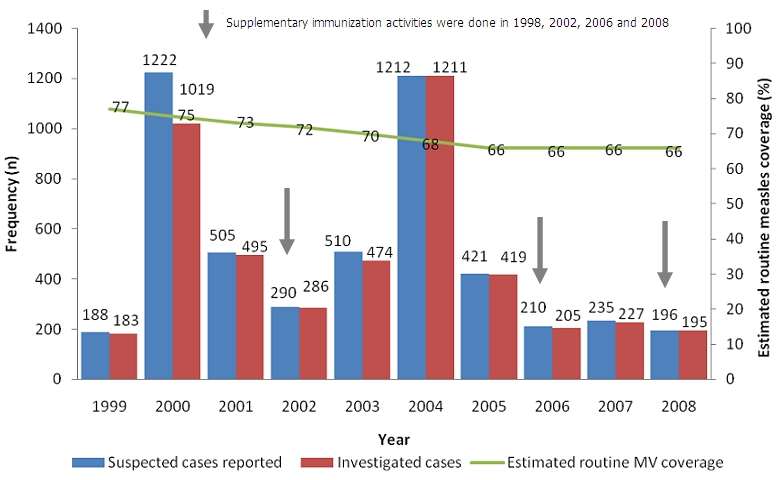
Number of reported suspected cases against number of cases investigated and estimated routine measles vaccine (MV) coverage per year, Jan 1999 - Dec 2000

Despite three supplemental immunization activities (SIAs) being carried out in Zimbabwe during the period 1999-2008, routine measles coverage has been declining according to WHO published estimates for the same period. This has been coupled with a decline in yearly reported suspected measles cases with exceptions recorded in the years 2000 and 2004. The proportion of investigated cases among the suspected for the same period however improved with at least 90% of cases investigated yearly (Figure 1).

Characterization of specimens received at the laboratory yearly during the period under review revealed that the proportion of specimens arriving at the laboratory timely (within 3 days of being taken) was consistently below the WHO recommended target of 80% in the period 1999-2008 with the exception of 2008. The proportion of serum specimens arriving at the national measles virology laboratory in good condition during the period 2004-2008 was below the WHO recommended target of 90 % except for the years 2005 and 2008. For the period 1999-2003, no data was captured on nature of specimens that were being collected and sent to the laboratory. The proportion of specimens received at the laboratory with results sent to the national level timely (within 7 days of specimen reciept at the laboratory) was below the recommended WHO target of 80% throughout 1999-2008. However, a general increase in proportion of specimens with results sent out timely from the laboratory was noted in the period 1999-2005. This was followed by a decline in the period 2005-2007 and then an increase in 2008 (Table 2).

**Table 2:** Characteristics of Specimens Received at the Laboratory and Feedback of Results to National Level During the Period 1999 – 2008

Year	Number of Reported Suspected Cases	Proportion of Cases Investigated % (n)	Proportion of Specimens arriving at Lab within 3 days % (n)	Proportion of specimens received at Lab in Good Condition % (n)	Proportion of Specimens with Results sent to National Level within 7 days of Receipt at Lab % (n)
**1999**	188	97.3 (183)	**64.5** (118)	**0.0** (0)	**4.4** (8)
**2000**	1222	83.4 (1019)	**56.5** (576)	**0.0** (0)	**9.7** (99)
**2001**	505	98.0 (495)	**39.4** (195)	**0.0** (0)	**30.1** (149)
**2002**	290	98.6 (286)	**58.0** (166)	**0.0** (0)	**16.8** (48)
**2003**	510	92.9 (474)	**43.0** (204)	**0.0** (0)	**9.8** (46)
**2004**	1212	99.9 (1211)	**58.9** (713)	**65.4** (792)	**76.8** (930)
**2005**	421	99.5 (419)	**59.6** (250)	90.7 (380)	**78.9** (330)
**2006**	210	97.6 (205)	**66.7** (137)	87.1 (179)	**58.3** (120)
**2007**	235	96.6 (227)	**70.2** (159)	**60.9** (138)	**19.2** (44)
**2008**	196	99.5 (195)	82.1 (160)	94.9 (185)	**65.3** (127)

Bold highlight indicates failure to meet recommended WHO target on respective indicators

The proportion of investigated cases confirmed to be measles by serological investigation were within the WHO recommended target of less than 10%, during the period 1999-2008 except in 2003. The majority of investigated cases were confirmed to be rubella, a differential diagnosis of measles, with proportions ranging between 12-40%, during the period 1999-2008.

**Figure 2:**
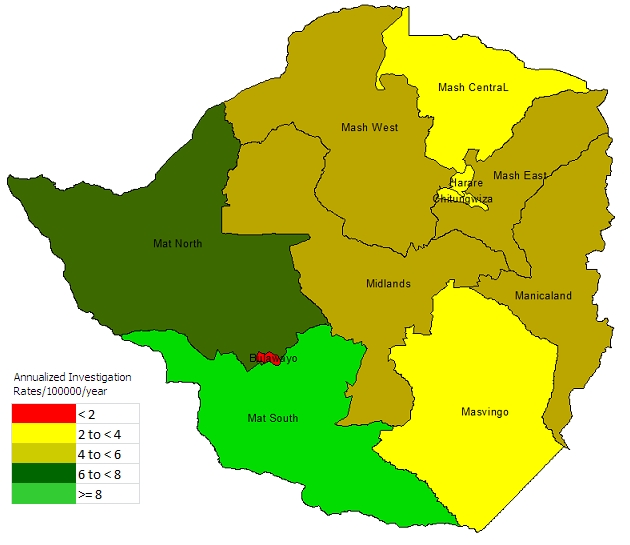
Average annualized rates of investigation (per 100 000 per year) by province for the period Jan 1999 - Dec 2008

Overall, the national measles surveillance system performed very well for the period 1999-2008, having an average annualized rate of investigation of 4 cases per 100 000 per year. On disaggregation of rates of case investigation per province on average, Matabeleland South and Matabeleland North Provinces performed extremely well in case investigation compared to the other provinces, while on the other hand Bulawayo Province performed badly (Figure 2).

**Figure 3:**
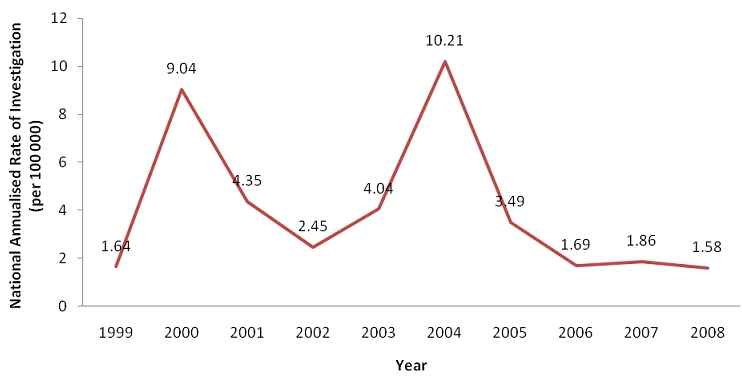
Trends of national annualised rates of investigation (per 100 000) for the period 1999 - 2008

On further analysis of the performance of the national measles case based surveillance system in terms of case investigation by year, there was generally a declining trend in the national annualized rates of investigation which decreased from 9.04 cases per 100,000 population per year in 2000 to 1.58 cases/100,000 population per year in 2008. The national surveillance system performed below the recommended WHO target of 2 cases/100,000 population per year for the period 2006-2008 (Figure 3).

## Discussion

Despite limitations in the nature of study design in that we were relying on already collected data and could not come up with definitive reasons as to why there were certain trends and anomalies in our findings, our study revealed a number of important findings. These were relating to quality of data collected by the surveillance system, populations still at risk of contracting measles in Zimbabwe and how the national measles case- based surveillance system has been performing in the period under review.

A significant proportion of the data had duplications and a small proportion had missing information on important variables (i.e. demographics and vaccination status) which made meaningful review of some of these records difficult. This is an indication of important gaps in the data collection system which needs urgent redress if reliable data interpretation is to be achieved and properly guided sound health policies are to be made based on the true picture on the ground. According to the Centre for Disease Control updated guidelines for evaluation of public health surveillance systems of 2001, quality of data is cited to be influenced by the performance of the screening and diagnostic tests (i.e. case definition) for the health-related event, the clarity of hardcopy or electronic surveillance forms, the quality of training and supervision of persons who complete these surveillance forms, and the care exercised in data management [9].

This study demonstrated that most affected age groups were the under 15 years, with 53.4% of them being children between the ages of 5-14 years, while the under 1 year and the above 15 year olds constituted the minority of cases. Similar observations were noted in other African countries in which an average 50% of cases occur in children aged 5-14 years [10].

This highlights the fact that the 9 months-15 year age group still remains a risky population for contracting measles in Zimbabwe and interventions like immunization campaigns need to be targeted at this group if morbidity and mortality due to measles are to be drastically reduced. In surveys carried out by the WHO in Malawi, mass campaign targeting of the 9 months-14 years age group was noted to reduce measles morbidity and mortality to near zero [11].

Overall, reported suspected and confirmed measles cases has been declining for the period 1999-2008 which may be attributed to among other things the successful EPI and Vitamin A supplementation as reported yearly by the MOHCW [12]. Discrepancies noted in the general declining trend in reported suspected and confirmed measles cases for the years 2000 and 2004, may be attributed to measles outbreaks that were reported in the same years in Zimbabwe. During these outbreaks, due to the overwhelming number of cases reported most cases i.e. suspected or probable could have been done on clinical basis which may also explain the sharp increase in cases reported.

Declining reported cases may also be reflective of the deteriorating health delivery that the country has been experiencing since the year 2000 [13, 14], which may be resulting in reduced capacity to detect and confirm measles cases. The associated decline in annualized rates of investigation noted since the year 2000 further supports the fact that health personnel were not doing enough in terms of actively investigating for suspected measles cases. This may have resulted in under reporting of a significant amount of cases. This is consistent with findings noted by the World Health Organisation in its proposal to respond to and control the ongoing measles outbreak in Zimbabwe in 2009, in which socio-economic challenges which were resulting in high staff attrition, closure of health facilities, shortages in gas supply impacting on cold chain functionality, lack of fuel & vehicles shortages were noted as major contributors to the decline in routine immunization coverage [13–15].

Challenges in maintaining the reverse cold chain (as suggested by a significant proportion of specimens arriving at the laboratory in bad condition) and communication and transport challenges (as suggested by a significant proportion of specimens failing to meet set timeframes in collection, receipt at the laboratory and feedback of results) may also explain why sensitivity of the system is low or on the decline. In an evaluation done by Chadambuka et al (2006) in Mberengwa, similar contributing factors were cited as reasons for poor performance of the measles case based surveillance system in that district [16].

## Conclusion

The national measles case based surveillance system improved in timeliness and feedback but its sensitivity declined due to reduced capacity to detect and confirm measles. The system has been operating below the recommended WHO standards. Data quality has on the overall been poor.

## Recommendations

We recommended that regular training and refresher courses be organized on EPI surveillance to improve health staff's knowledge of the system and hence the system's performance. There is need to conduct periodic reviews of provincial EPI surveillance data in order to strengthen weak districts. More research to determine why some provinces/districts are continually performing poorly should be facilitated.
